# Power without limits: exploring emotional regulation, moral authority, and psychiatric overreach through the *Wizarding World* of *Harry Potter*

**DOI:** 10.3389/fpsyt.2026.1889488

**Published:** 2026-07-08

**Authors:** Julio Torales, Gladys Estigarribia, Iván Barrios, Marcelo O’Higgins, Alcides de Jesús Sosa Gamarra, Fernando Ismael Da Silva Sánchez, João Mauricio Castaldelli-Maia, Antonio Ventriglio

**Affiliations:** 1 Facultad de Medicina, Grupo de Investigación en Salud Mental, Universidad Santa Clara de Asís, Caaguazú, Paraguay; 2 Facultad de Ciencias Médicas, Grupo de Investigación sobre Epidemiología de los Trastornos Mentales, Psicopatología y Neurociencias, Universidad Nacional de Asunción, San Lorenzo, Paraguay; 3 Vicerrectoría de Investigación y Postgrado, Universidad de Los Lagos, Osorno, Chile; 4 Facultad de Ciencias Médicas, Filial Santa Rosa del Aguaray, Cátedra de Bioestadística, Universidad Nacional de Asunción, Santa Rosa del Aguaray, Paraguay; 5 Department of Psychiatry, University of São Paulo, São Paulo, Brazil; 6 Department of Clinical and Experimental Medicine, University of Foggia, Foggia, Italy

**Keywords:** emotional regulation, Harry Potter, narrative medicine, psychiatric ethics, psychiatric overreach

## Abstract

**Background:**

Psychiatry has never had more tools to relieve psychological suffering. Psychopharmacology, neuromodulation, early intervention, digital monitoring, and preventive approaches have expanded what clinicians can do. Yet this growing technical capacity also raises an uneasy question: when does care become control? Cultural narratives can offer spaces for examining such tensions, particularly when they dramatize power, vulnerability, suffering, and moral authority.

**Aim:**

This narrative review uses the *Wizarding World* of *Harry Potter* as a conceptual lens to examine emotional regulation, moral authority, and psychiatric overreach, focusing on two contrasting figures: Lord Voldemort and Isidora Morganach.

**Methods:**

A theory-informed narrative review was conducted using a concept-focused analytic framework and structured conceptual synthesis. Literature was identified through focused searches in PubMed, PsycINFO, Scopus, and Google Scholar from database inception to June 2026, complemented by backward reference tracking. Sources covered *Harry Potter* scholarship, emotional regulation, psychological flexibility, narcissism and Dark Triad traits, medicalization, overdiagnosis, clinical ethics, and narrative medicine. To improve transparency, the synthesis distinguished direct literature on the *Wizarding World* from indirect psychiatric, psychological, ethical, and medical humanities scholarship used for conceptual interpretation.

**Results:**

Two pathways to psychiatric overreach and an intermediate zone of proportionate care were identified. The first, represented by Voldemort, involves destructive domination, emotional suppression, denial of vulnerability, instrumental use of others, and coercive authority. The second, represented by Isidora Morganach, involves hyper-compassionate control, intolerance of suffering, elimination of negative affect, moral certainty, and paternalistic intervention. Between these extremes, the intermediate zone emphasizes emotional integration, psychological flexibility, shared decision-making, contextual judgment, ethical proportionality, and clinical humility. Although one pathway is cruel and the other compassionate, both illustrate power exercised without sufficient emotional integration or ethical restraint.

**Conclusion:**

By exploring psychiatric questions through the *Wizarding World*, this review highlights how narrative frameworks can clarify the ethical boundaries of intervention. The central lesson is not that intervention is dangerous, but that intervention must remain proportionate, reflective, and ethically bounded. Psychiatry’s task is not to abolish all distress, but to relieve suffering while preserving autonomy, meaning, and human vulnerability.

## 1 Introduction

Over the past two decades, psychiatry has increasingly looked beyond traditional clinical texts to engage with cultural narratives as spaces for reflection. Fictional worlds, when taken seriously, can illuminate ethical tensions that clinical manuals alone cannot fully capture. They offer structured moral landscapes in which power, suffering, vulnerability, and authority unfold in recognizably human ways. The *Harry Potter* universe is one such landscape. It has already attracted scholarly attention in mental health and psychological contexts, from its use in adolescent psychotherapy (1), to its role in recovery narratives and meaning-making (2), to experimental research demonstrating measurable emotional engagement during reading (3), and evidence that identification with its characters may influence social attitudes and prejudice (4). Even the so-called “Dobby effect” has entered psychological research as a metaphor for guilt-related self-punishment (5). Taken together, this body of work suggests that the *Wizarding World* can function as more than popular entertainment; it can serve as a conceptual lens for examining clinically relevant questions.

In this review, the term *Wizarding World* refers to the broader narrative universe established by J. K. Rowling’s original *Harry Potter* novels (6) and subsequently expanded through films and officially licensed works. This expanded canon includes the video game *Hogwarts Legacy* (7), set in the nineteenth century within the same fictional universe. While the original series centers on the rise and fall of Lord Voldemort, later narrative developments introduce additional figures whose moral complexity extends the thematic architecture of the world. Among them is Isidora Morganach, a character appearing in *Hogwarts Legacy* rather than in the primary literary canon. Although not part of the original novels, her storyline deepens a recurring tension within the *Wizarding World*: the uneasy boundary between relieving suffering and exercising control.

The inclusion of Isidora Morganach requires methodological clarification because she belongs to an officially licensed transmedia extension rather than to the original literary canon. Contemporary transmedia storytelling often develops narrative worlds across multiple media, allowing later works to expand themes, characters, and moral conflicts within an established fictional universe (8, 9). In this review, *Hogwarts Legacy* is not treated as equivalent to the original novels in terms of authorship, literary status, or canonical centrality. Rather, it is used as an expanded narrative setting that preserves the broader thematic architecture of the *Wizarding World*, including magic, power, memory, vulnerability, suffering, and moral choice. Isidora is therefore included not as evidence of authorial intention or as a primary-canon figure, but because her storyline provides a particularly clear narrative configuration of benevolent overreach: the attempt to relieve suffering by removing it. This complements Voldemort’s configuration of domination without empathy and allows the review to examine two contrasting forms of power without emotional integration.

The use of the *Wizarding World* in this review does not depend on J. K. Rowling having clinical training, nor does it treat authorial intention as psychiatric evidence. Rather, the relevance of the narrative lies in its cultural reach, symbolic density, and capacity to represent recognizable human experiences such as fear, grief, vulnerability, power, moral choice, and the wish to relieve suffering. This approach is consistent with narrative medicine and medical humanities, where literary and fictional texts are not used because their authors possess clinical authority, but because narratives can help clinicians and learners reflect on meaning, subjectivity, ethical ambiguity, and the lived dimensions of suffering (10, 11, 12). In this sense, fictional characters are not treated as clinical cases or diagnostic subjects; they are used as symbolic configurations through which broader psychological and ethical tensions can be examined.

These narrative tensions resonate with a contemporary psychiatric dilemma. Psychiatry has never possessed greater technical capacity to influence mood, cognition, and behavior. Psychopharmacology, neuromodulation, preventive strategies, early intervention models, digital monitoring, and structured risk management have expanded the scope of possible action. These developments have brought real clinical benefits, particularly for people with severe or disabling mental disorders. Yet they have also intensified long-standing concerns about diagnostic expansion, overdiagnosis, and the medicalization of ordinary emotional life (13–16). Examples of psychiatric intervention exceeding appropriate limits include the overdiagnosis of conditions such as attention-deficit/hyperactivity disorder in children and adolescents (17), the transformation of contextually understandable sadness into depressive disorder (13), the use of coercive practices such as seclusion and restraint despite their ethical and clinical risks (18, 19), and the expansion of early detection or digital monitoring approaches without sufficient attention to consent, uncertainty, stigma, privacy, or proportionality (20–23). These examples do not imply that psychiatric intervention is inherently problematic. Rather, they show that even clinically justified tools may become ethically unstable when applied too broadly, too early, too coercively, or without sufficient attention to context and patient agency. The profession therefore faces a delicate balance: alleviating genuine suffering without redefining all distress as pathology. The question is no longer simply whether we can intervene, but how far intervention should extend.

Emotional regulation theory provides a useful framework for thinking through this boundary. Regulation does not imply the absence of negative affect; rather, it refers to the modulation of emotional experience, expression, and physiological response in ways that remain adaptive (24–26). Empirical evidence indicates that rigid suppression strategies are associated with poorer psychological outcomes across disorders, whereas flexible regulation and psychological flexibility are linked to better adaptation and resilience (27–29). Acceptance-based approaches similarly emphasize changing one’s relationship to internal experience rather than eliminating distress itself (30). In clinical settings, this distinction matters because fear, sadness, grief, and distress may be painful without being inherently pathological; their clinical meaning depends on severity, persistence, impairment, context, and the person’s own values and goals (13, 16).

Within this conceptual frame, the figure of Lord Voldemort can be read as an extreme narrative representation of grandiosity coupled with the denial of vulnerability. Contemporary dimensional models describe narcissism along a spectrum, with pathological forms characterized by entitlement, dominance, antagonism, and diminished empathy (31, 32). Traits associated with the Dark Triad (narcissism, psychopathy, and Machiavellianism) have been linked to manipulative interpersonal styles, callousness, and instrumental use of others (33–35). In narrative terms, Voldemort embodies a configuration in which emotional suppression, rejection of vulnerability, and instrumentalization of others consolidate into coercive authority.

By contrast, Isidora Morganach represents a different configuration of power without limits. Her project is not domination but relief: she seeks to remove emotional pain entirely. Yet this narrative arc can be supported conceptually by evidence on experiential avoidance, distress intolerance, and psychological inflexibility. Distress tolerance refers to the perceived or actual capacity to withstand negative psychological states, whereas experiential avoidance involves attempts to escape or eliminate unwanted internal experiences even when such avoidance produces longer-term costs (30, 36). From this perspective, the attempt to eradicate suffering rather than regulate or integrate it risks collapsing the adaptive functions of negative affect and may resemble, in exaggerated fictional form, real clinical concerns about overmedicalization, overdiagnosis, paternalism, and excessive intervention (13–15, 37, 38). Compassion, when detached from autonomy, proportionality, and ethical restraint, may also overstep.

The contrast between destructive domination and hyper-compassionate control converges on a shared structural concern: the exercise of authority without sufficient emotional integration or ethical restraint. One configuration treats vulnerability as weakness; the other treats it as a defect to be corrected. Both illuminate risks relevant to contemporary psychiatric practice. The unique contribution of this review is not to propose a new ethical principle or to claim that fictional narratives provide clinical evidence. Rather, the *Wizarding World* is used as an analytic and pedagogical device that places two forms of psychiatric overreach in conceptual relation: domination without empathy and compassion without restraint. This framework highlights that excessive clinical power may arise not only from coercion, grandiosity, or authoritarian control, but also from benevolent motives when the wish to relieve suffering becomes detached from autonomy, proportionality, and emotional integration. This narrative review therefore uses the *Wizarding World* as a structured lens through which to examine emotional regulation, moral authority, and the boundaries of psychiatric intervention. The aim is not to diagnose fictional characters, but to clarify a professional question that remains deeply real: how can psychiatry exercise its expanding capacities without allowing care to become control?

## 2 Methods

### 2.1 Review design

This article was conducted as a theory-informed narrative review with a concept-focused analytic framework. Narrative methodology was selected because the literature relevant to emotional regulation, moral authority, psychiatric overreach, and cultural representations of mental health is heterogeneous in disciplinary origin, conceptual language, methodological design, and outcome focus, limiting the feasibility of systematic aggregation or quantitative meta-analytic synthesis (39, 40).

The aim of this review was to synthesize theoretical models, empirical findings, ethical perspectives, and narrative-clinical parallels relevant to the boundaries of psychiatric intervention. The review follows established guidance for narrative literature syntheses while incorporating structured evidence summarization to improve methodological transparency (39–41).

To reduce unstructured interpretative bias, the final synthesis was organized around predefined conceptual domains rather than an open-ended essayistic approach. These domains included *Harry Potter* and mental health scholarship; emotional regulation and psychological flexibility; narcissism, dominance, and Dark Triad traits; psychiatric overreach and medicalization; ethics, paternalism, and clinical authority; and narrative medicine and medical humanities. Within these domains, we distinguished direct evidence, defined as scholarship explicitly engaging with the *Wizarding World*, from indirect evidence, defined as psychiatric, psychological, ethical, and medical humanities literature used to support conceptual interpretation. This procedure was not intended to reproduce the methods of a systematic review, but to make explicit the evidentiary basis from which the conceptual framework was developed.

Particular emphasis was placed on integrating psychiatric, psychological, ethical, and medical humanities perspectives into a unified conceptual framework centered on emotional regulation, vulnerability, power, and intervention.

### 2.2 Literature search strategy

A focused literature search was conducted using PubMed, PsycINFO, and Scopus as primary databases, from database inception to June 2026. Additional targeted searches were performed through Google Scholar to identify influential conceptual papers, foundational theoretical works, and highly cited publications in medical humanities and narrative psychiatry.

Search terms included combinations of concepts related to the *Harry Potter* universe and contemporary psychiatric theory, including “Harry Potter,” “Wizarding World,” “Hogwarts Legacy,” “fiction psychiatry,” “medical humanities,” “narrative medicine,” “narrative psychiatry,” “emotional regulation,” “emotion suppression,” “psychological flexibility,” “experiential avoidance,” “distress tolerance,” “pathological narcissism,” “Dark Triad,” “grandiosity,” “medicalization,” “overdiagnosis psychiatry,” “psychiatric overreach,” “medical paternalism,” “clinical authority,” and “ethics psychiatry.”

The full search strings, including Boolean operators, are provided in the Supplementary Material as **Supplementary Table 1**. Because the review was concept-focused and interdisciplinary rather than a systematic review, no formal MeSH analyzer or automated synonym-generation tool was used. Search terms were developed iteratively from the main conceptual domains of the review, including narrative medicine, emotional regulation, psychological flexibility, narcissism and Dark Triad traits, medicalization, overdiagnosis, paternalism, psychiatric ethics, and the *Wizarding World*. Controlled vocabulary and database indexing terms were considered when available, but the final search strategy relied primarily on keyword combinations to capture literature across psychiatry, psychology, ethics, and medical humanities.

Searches were limited to peer-reviewed articles and scholarly books published in English. Priority was given to systematic reviews, meta-analyses, randomized and experimental studies, theoretical models, and influential conceptual papers directly relevant to psychiatry, psychology, emotional regulation, narcissism, medicalization, and ethics.

Given the interdisciplinary nature of the topic, selected books and conceptual works from medical humanities and narrative medicine were also included when they contributed substantially to the interpretative framework of the review.

Backward reference tracking of key reviews and foundational theoretical papers was performed to ensure coverage of influential models and frequently cited conceptual contributions.

### 2.3 Study selection and data extraction

Sources were selected based on relevance to one or more of the following conceptual domains:

1. scholarly work using the *Harry Potter* universe in psychological, psychiatric, therapeutic, educational, or mental health contexts;
2. empirical and theoretical literature on emotional regulation, emotion suppression, experiential avoidance, psychological flexibility, and distress tolerance;
3. literature on narcissism, grandiosity, Dark Triad traits, dominance, and interpersonal instrumentalization;
4. conceptual and clinical literature addressing psychiatric overreach, medicalization, overdiagnosis, paternalism, and ethical boundaries in psychiatric practice;
5. scholarship on transmedia storytelling and expanded narrative worlds relevant to the methodological use of officially licensed adaptations within broader fictional universes.
6. scholarship in narrative medicine, medical humanities, and narrative psychiatry relevant to the interpretation of fictional narratives in healthcare contexts.

Eligibility was applied through a concept-driven screening process. Candidate sources were first assessed at the level of title, abstract, keywords, or bibliographic information to determine whether they addressed one of the predefined domains. Sources considered potentially relevant were then examined in full text, when available, to determine whether they made a substantive empirical, theoretical, ethical, or interpretative contribution to the review question. Sources were included when they contributed directly to the use of the *Wizarding World* as a psychological or psychiatric narrative framework, or indirectly to the conceptual interpretation of emotional regulation, vulnerability, grandiosity, distress intolerance, medicalization, overdiagnosis, paternalism, coercion, clinical authority, or narrative medicine. Sources were excluded when they were non-scholarly, purely fan-based or journalistic, unrelated to psychiatric, psychological, ethical, or medical humanities constructs, or when they mentioned fictional material only descriptively without contributing to the conceptual questions of the review.

Because this was a narrative review rather than a systematic review, article selection was not intended to be exhaustive and no formal risk-of-bias assessment or pooled evidence grading was performed. However, explicit domains, eligibility criteria, source types, and analytic distinctions were used to improve transparency and reduce unstructured interpretative bias. Selected sources were then categorized according to their primary contribution to the conceptual framework. Sources were not classified as belonging literally to one fictional character or another; rather, Voldemort and Isidora Morganach were treated as heuristic narrative poles through which broader domains of evidence were interpreted.

For each included source, information was extracted regarding publication type, disciplinary domain, principal construct addressed, methodological design when applicable, key findings or theoretical contributions, and overall relevance to the conceptual framework of the review. We also recorded whether each source provided direct evidence related to the *Wizarding World* or indirect evidence supporting one of the psychiatric, psychological, ethical, or medical humanities domains of the synthesis.

Extracted information was organized into structured summary tables to enhance transparency regarding literature domains, conceptual contributions, and narrative-clinical parallels (**Tables 1**, **2**).

**Table 1:** Main literature domains included in the conceptual synthesis.

Domain	Key references	Main contribution to the review
Harry Potter and mental health scholarship	Noctor (1); Tribe et al. (2); Vezzali et al. (4); Hsu et al. (3); Nelissen & Zeelenberg (5)	Supports the use of the *Wizarding World* as a psychologically and clinically relevant narrative framework
Emotional regulation and psychological flexibility	Gross (24, 25); Aldao et al. (27); Kashdan & Rottenberg (28); Hayes et al. (30)	Distinguishes adaptive regulation from emotional suppression and experiential avoidance
Narcissism, dominance, and Dark Triad traits	Krizan & Herlache (31); Miller et al. (32); Paulhus & Williams (34); Jones & Figueredo (33); Wissing & Reinhard (35)	Provides dimensional models of grandiosity, dominance, and interpersonal instrumentalization
Psychiatric overreach and medicalization	Wakefield (16); Horwitz & Wakefield (13)Moynihan et al. (14); Paris (15); Frances (42)	Frames concerns regarding diagnostic expansion, overmedicalization, and excessive intervention
Ethics, paternalism, and clinical authority	Beauchamp & Childress (37); Emanuel & Emanuel (38); Appelbaum (43)	Supports discussion regarding moral authority, autonomy, and therapeutic boundaries
Transmedia storytelling and expanded narrative worlds	Jenkins (8); Scolari (9)	Supports the methodological legitimacy of considering officially licensed adaptations as expansions of a narrative world, while distinguishing them from the original literary canon
Narrative medicine and medical humanities	Charon (10); DasGupta & Charon (11); Holmes (12); Strawson (44); Woods (45)	Supports and critically delimits the interpretative use of narrative frameworks in psychiatric reflection, including their value for meaning-making and ethical analysis as well as their methodological limitations.

**Table 2:** Narrative-clinical parallels within the *Wizarding World*.

Narrative element	Character/narrative example	Clinical or conceptual parallel	Psychiatric relevance
Denial of vulnerability and finitude	Lord Voldemort	Grandiosity, emotional suppression, and refusal of vulnerability	Illustrates coercive authority, maladaptive regulation, and the pursuit of invulnerability
Instrumental use of others	Voldemort and followers	Dark Triad interpersonal traits	Highlights dominance-based relational patterns
Control through fear	Voldemort’s regime	Authoritarian misuse of power	Reflects risks associated with power detached from empathy
Elimination of emotional pain	Isidora Morganach	Intolerance of distress and emotional overcontrol	Reflects risks of excessive intervention
Extraction of negative emotion	Ancient magic in Hogwarts Legacy	Emotional suppression vs integration	Raises questions regarding the adaptive role of distress
Hyper-compassionate intervention	Isidora Morganach	Paternalism and psychiatric overreach	Illustrates how benevolent intentions may exceed ethical boundaries

### 2.4 Conceptual synthesis framework

Given the heterogeneous nature of the evidence base, an explicit distinction was maintained between direct and indirect evidence throughout the analysis.

Direct evidence refers to scholarship explicitly examining the *Harry Potter* universe in relation to psychology, psychotherapy, emotional engagement, prejudice reduction, mental health recovery, or educational processes.

Indirect evidence refers to broader psychiatric, psychological, ethical, and medical humanities literature that does not directly address the *Wizarding World* but informs the interpretation of emotional regulation, vulnerability, narcissism, authority, and intervention.

The inclusion of indirect evidence does not imply that fictional characters can be treated as clinical subjects. Rather, such literature was used to construct a conceptual framework for examining narrative representations of power, suffering, emotional control, and psychiatric authority. Extrapolation was therefore limited to thematic and conceptual parallels rather than diagnostic claims.

To further reduce the risk of category confusion, the analysis distinguished three levels of interpretation. First, narrative descriptions refer to events, actions, and character arcs within the fictional world. Second, conceptual parallels identify analogies between these narrative patterns and constructs from psychiatry, psychology, ethics, or medical humanities. Third, broader psychiatric claims are made only when supported by independent scholarly literature and refer to clinical practice or psychiatric ethics in general, not to the fictional characters themselves. Terms such as narcissism, grandiosity, emotional suppression, distress intolerance, and paternalism are therefore used as interpretive constructs for analyzing narrative configurations of power, vulnerability, and intervention, not as diagnostic labels or clinical formulations applied to Voldemort or Isidora Morganach.

## 3 Results

The final reference set comprised 58 sources. Of these, 53 scholarly sources informed the substantive conceptual synthesis, including transmedia storytelling scholarship and methodological critiques of narrative medicine, three sources supported the narrative review methodology, and two primary narrative sources provided the fictional material analyzed in the review.

As summarized in **Tables 1** and **2**, the included sources were organized according to their primary conceptual contribution to the synthesis and their relevance to the narrative-clinical parallels examined in this review. The substantive synthesis included direct scholarship on the *Harry Potter* universe in psychological and mental health contexts, transmedia storytelling scholarship, and indirect psychiatric, psychological, ethical, and medical humanities literature. Direct sources supported the use of the *Wizarding World* as a culturally recognizable narrative framework, particularly through work on psychotherapy, recovery, emotional engagement, prejudice reduction, and guilt-related self-punishment (1–5). Transmedia scholarship provided the methodological rationale for considering officially licensed adaptations as expansions of a narrative world rather than as equivalent to the original literary canon (8, 9).

Indirect sources were grouped according to their primary conceptual contribution. Literature on narcissism, Dark Triad traits, moral disengagement, emotional suppression, and coercion informed the destructive domination pathway, whereas literature on distress intolerance, experiential avoidance, medicalization, overdiagnosis, paternalism, and excessive intervention informed the hyper-compassionate control pathway. Sources addressing emotional regulation, psychological flexibility, shared decision-making, clinical ethics, and clinical humility informed the intermediate zone of proportionate psychiatric care.

Narrative medicine and medical humanities sources supported the interpretative and pedagogical use of fictional narratives for psychiatric reflection, while critiques of narrativity helped define the methodological limits of this approach (10–12, 44, 45). Thus, sources were not assigned to characters as if Voldemort or Isidora Morganach were clinical categories; rather, the characters were used as heuristic narrative anchors for broader domains of psychiatric and ethical literature.

### 3.1 The *Wizarding World* as a psychiatric narrative framework

The *Wizarding World* offers a useful narrative framework for examining psychiatric questions because it repeatedly dramatizes the relationship between power, vulnerability, suffering, and moral choice. Previous scholarship has already shown that the *Harry Potter* universe can be used meaningfully in mental health-related contexts, including adolescent psychotherapy, recovery narratives, affective engagement during reading, prejudice reduction, and guilt-related self-punishment (1–5). This literature supports the use of the narrative world not as a substitute for clinical evidence, but as a culturally recognizable symbolic field through which psychiatric and psychological constructs can be explored.

The synthesis identified two recurring configurations of power and emotional regulation within this narrative field, rather than two diagnostic profiles. The first configuration centers on domination, suppression of vulnerability, instrumental relationships, and coercive authority. The second centers on the elimination of suffering, paternalistic intervention, moral certainty, and the risk of compassion exceeding ethical limits. Within the *Wizarding World*, Voldemort and Isidora Morganach provide recognizable narrative anchors for these configurations, while the intermediate space between them represents proportionate psychiatric care guided by emotional integration, autonomy, clinical humility, and contextual judgment.

This contrast is clinically relevant because contemporary psychiatry also operates within a field of expanding technical capacity. The ability to diagnose, treat, prevent, and modulate psychological distress has increased substantially, but this expansion has also generated concern regarding overdiagnosis, medicalization, and the risk of exceeding the therapeutic mandate (13–16, 42). In this sense, the narrative contrast is not the rationale for the review itself, but a conceptual device for examining a real psychiatric problem: how can suffering be relieved without transforming care into control?

### 3.2 Destructive domination and emotional suppression: Lord Voldemort

Lord Voldemort can be read as a narrative representation of power organized around grandiosity, dominance, and the refusal of vulnerability. This interpretation is conceptual rather than diagnostic: the cited literature is used to illuminate a narrative configuration of power, vulnerability, and emotional suppression, not to assign a psychiatric disorder to the character. Contemporary dimensional models of narcissism describe pathological narcissism not as a single fixed category, but as a spectrum involving grandiosity, entitlement, antagonism, dominance, and impaired empathy (31, 32). The Dark Triad literature similarly describes a constellation of narcissism, Machiavellianism, and psychopathy associated with manipulative interpersonal strategies, callousness, and instrumental use of others (33–35). Voldemort’s narrative role aligns with this extreme configuration: relationships are subordinated to control, loyalty is maintained through fear, and others are valued primarily as instruments of power.

A central feature of this pathway is the denial of vulnerability. Voldemort’s fear of death is not integrated into a broader human experience of limitation, dependency, or mourning. Instead, it becomes the organizing principle of domination. From the perspective of emotional regulation theory, this resembles a pathological form of suppression: rather than acknowledging fear as an affective signal, the character attempts to eliminate vulnerability through external control and magical fragmentation. Emotion regulation research has repeatedly distinguished adaptive modulation of emotion from rigid suppression, with suppression generally associated with poorer psychological and interpersonal outcomes (24, 25, 27).

The Horcruxes provide a particularly strong symbolic representation of this refusal of vulnerability. They should not be interpreted clinically or literally, but narratively they embody a fantasy of survival through fragmentation. Rather than accepting mortality as part of human finitude, Voldemort divides the self in pursuit of invulnerability. This symbolic structure resonates with broader psychological literature on avoidance, emotional inflexibility, and the costs of refusing internal experience (28, 30). The result is not freedom from fear, but an increasingly coercive and dehumanized form of existence.

This pathway therefore illustrates how emotional suppression may become politically and relationally dangerous when combined with grandiosity and moral disengagement. Social psychological work on moral disengagement has shown how harmful conduct becomes easier when individuals cognitively reconstruct violence, minimize responsibility, or dehumanize others (46). In Voldemort’s case, fear of vulnerability is displaced into domination; emotional failure becomes social violence. The psychiatric relevance lies not in diagnosing the character, but in recognizing a narrative pattern in which unintegrated fear and grandiosity produce coercive authority.

### 3.3 Hyper-compassionate control and intolerance of suffering: Isidora Morganach

Isidora Morganach represents a different and more clinically subtle pathway. As with Voldemort, this reading is conceptual rather than diagnostic: Isidora is not treated as a clinical subject, but as a narrative configuration through which the review examines benevolent overreach, emotional overcontrol, and the ethical risks of eliminating suffering. Unlike Voldemort, her project is not organized around domination for its own sake. It begins with the desire to relieve suffering. In *Hogwarts Legacy*, Isidora discovers that she can remove emotional pain from others through Ancient Magic, initially presenting this intervention as a compassionate response to distress (7). This narrative arc is especially relevant to psychiatry because it raises a question at the heart of clinical practice: when does the wish to relieve suffering become an attempt to control the human experience of suffering itself?

From the perspective of emotion regulation, Isidora’s intervention collapses an important distinction between regulating emotion and eradicating emotion. Contemporary models emphasize that negative affect is not inherently pathological. Fear, sadness, grief, and distress may be painful, but they can also carry adaptive, relational, and meaning-making functions (24, 25). Clinical approaches grounded in psychological flexibility similarly argue that mental health depends not on the absence of difficult internal experiences, but on the capacity to relate to them in flexible and meaningful ways (28, 30). Isidora’s project bypasses this process: suffering is treated not as something to be understood, metabolized, or integrated, but as something to be extracted.

This pathway can also be understood through the lens of distress intolerance and experiential avoidance, although the formulation of “externally directed distress intolerance” should be understood as a conceptual extension proposed by this review rather than as an established diagnostic or psychometric construct. Distress tolerance refers to the perceived or actual capacity to withstand negative psychological states, whereas experiential avoidance involves attempts to escape or eliminate unwanted internal experiences, even when doing so produces long-term costs (30, 36). The present framework extends these ideas from the individual’s relationship to their own internal states to the helper’s response to the perceived suffering of another person. In Isidora’s case, the intolerable object is not only her own pain, but the pain of others. Her intervention reflects a form of externally directed distress intolerance: the suffering of another person becomes something she cannot bear, and therefore something she feels compelled to remove. This interpretation also resonates with psychotherapy literature on rescue fantasies, in which helping professionals may experience powerful wishes to rescue or repair the suffering of those they treat, requiring reflective awareness to prevent overinvolvement or boundary erosion (47).

Clinically, this distinction is important. Psychiatry and psychotherapy frequently aim to reduce suffering, but reduction is not the same as elimination. Overly aggressive intervention may unintentionally undermine autonomy, meaning-making, and emotional integration. This concern is reflected in debates about overdiagnosis, medicalization, and the treatment of normative distress as disorder (13–16). Isidora’s storyline dramatizes this concern in exaggerated form: compassion becomes ethically unstable when it assumes the authority to decide which emotions should exist.

The ethical problem is therefore not compassion itself, but compassion without boundaries. Clinical ethics has long emphasized that beneficence must be balanced with autonomy, non-maleficence, and respect for the person’s values and agency (37). Similarly, models of the physician–patient relationship distinguish paternalistic authority from more deliberative and interpretive approaches that preserve patient agency (38, 48). Isidora’s actions illustrate the risk of moral certainty: the helper becomes so convinced of the good of intervention that the subjectivity of the other is overridden.

### 3.4 Comparative synthesis: two pathways to psychiatric overreach and the intermediate zone of proportionate care

The comparison between Voldemort and Isidora suggests two distinct pathways through which power may exceed ethical limits. The first pathway, destructive domination, is organized around grandiosity, suppression of vulnerability, instrumental relationships, and coercive authority, a configuration supported conceptually by literature on narcissism, Dark Triad traits, moral disengagement, emotional suppression, and coercive practices (18, 19, 24, 25, 27, 31–34, 46). The second pathway, hyper-compassionate control, is organized around intolerance of suffering, elimination of negative affect, moral certainty, and paternalistic intervention, and is informed by literature on experiential avoidance, distress intolerance, medicalization, overdiagnosis, paternalism, and clinical ethics (13–16, 30, 36–38). These pathways differ in motivation but converge in outcome: both involve the exercise of power without adequate emotional integration.

In the first pathway, vulnerability is treated as weakness. Voldemort’s response to fear is domination. He does not seek to understand or regulate fear; rather, he attempts to abolish its consequences through control over others and magical efforts to achieve invulnerability. This reading is consistent with evidence that rigid suppression and psychological inflexibility are associated with poorer psychological and interpersonal outcomes (24, 25, 27, 28). In the second pathway, vulnerability is treated as an error to be corrected. Isidora’s response to suffering is removal. She does not accompany distress; she extracts it. This pathway parallels literature on experiential avoidance and distress intolerance, in which attempts to eliminate unwanted internal experience may produce longer-term costs (30, 36**).** Both trajectories therefore reject the ordinary human task of integrating painful affect.

This distinction helps clarify why psychiatric overreach should not be understood only as coercive or authoritarian. It may also emerge from benevolent motives. The history of psychiatry includes clear examples of coercive misuse of authority, but contemporary concerns about overreach also include subtler processes such as diagnostic inflation, premature intervention, excessive risk management, and the medicalization of distress that may be painful but not necessarily pathological (13, 15, 16, 42). Related concerns are also reflected in debates on overdiagnosis in child and adolescent psychiatry, coercive practices, and the ethical challenges of expanding digital and early-intervention approaches (17, 19–23). The *Wizarding World* allows these risks to be represented in narrative form through two contrasting moral extremes.

The central conceptual finding of this review is therefore that psychiatric overreach may arise through at least two routes. One route is domination without empathy; the other is compassion without restraint. Both are clinically relevant because psychiatric practice requires not only technical capacity, but also disciplined judgment about when intervention is appropriate, proportionate, and ethically justified, consistent with established principles of autonomy, beneficence, shared decision-making, and clinical humility (49, 37, 50, 38, 51).

Importantly, the two characters should not be understood as mutually exclusive diagnostic categories or as endpoints into which all reviewed papers were forced. They function as heuristic narrative poles. Sources were categorized according to their primary conceptual contribution rather than according to the fictional character to which they appeared closest. Literature on narcissism, Dark Triad traits, moral disengagement, coercion, and emotional suppression primarily informed the destructive domination pathway (18, 19, 24, 25, 27, 46, 31–34). Literature on distress intolerance, experiential avoidance, medicalization, overdiagnosis, paternalism, and excessive intervention primarily informed the hyper-compassionate control pathway (13–16, 30, 36–38, ). Sources addressing emotional regulation, psychological flexibility, shared decision-making, clinical ethics, and narrative medicine informed the intermediate space between these poles, where psychiatric care remains proportionate, reflective, collaborative, and ethically bounded (10–12, 28, 37, 49, 50).

**Figure 1:**
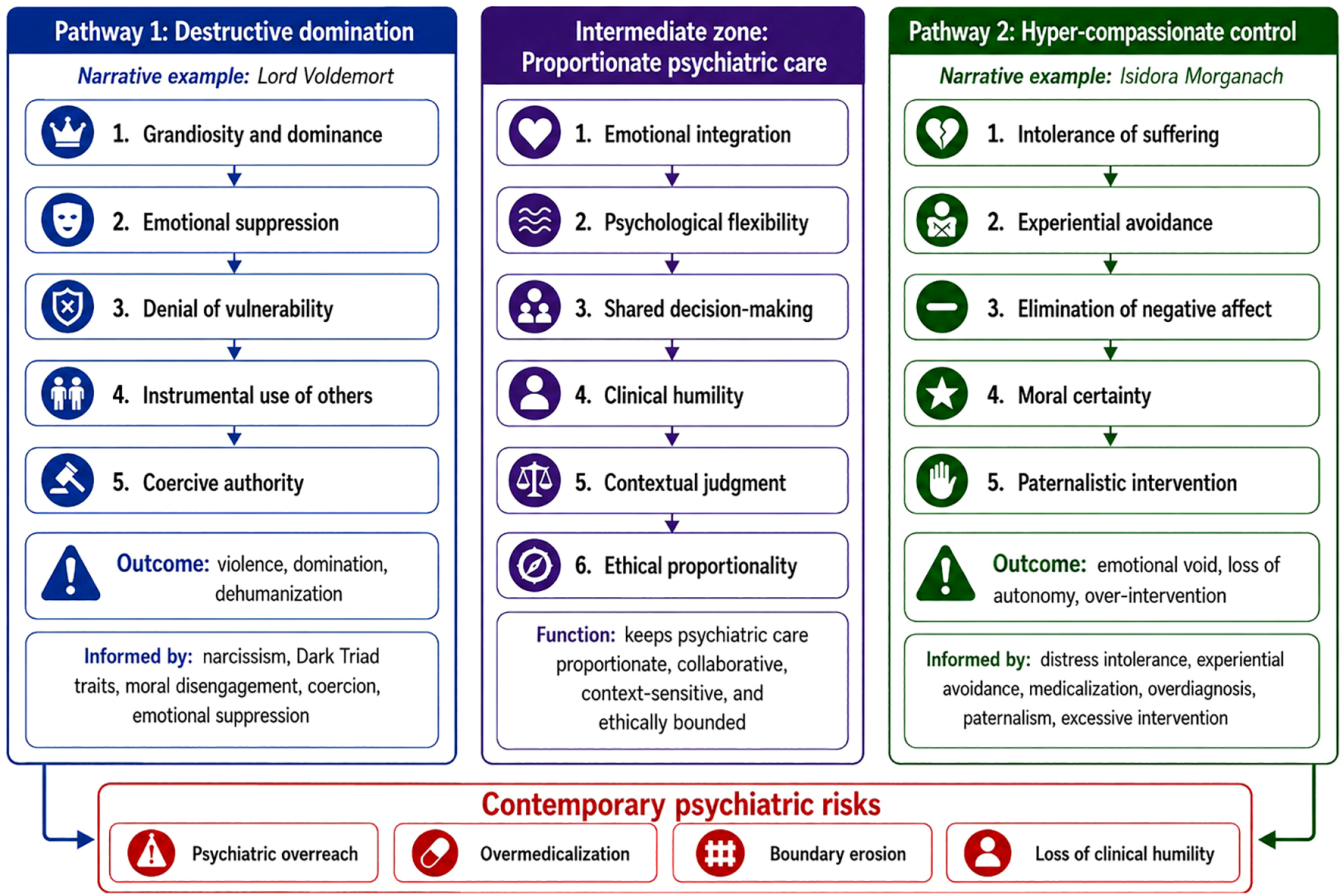
Power without emotional integration: heuristic pathways to psychiatric overreach and the intermediate zone of proportionate psychiatric care. This image presents the conceptual model developed in this review. Lord Voldemort and Isidora Morganach are used as heuristic narrative poles, not as diagnostic categories. The left pathway, anchored by Voldemort, represents destructive domination, characterized by grandiosity, emotional suppression, denial of vulnerability, instrumental use of others, and coercive authority. The right pathway, anchored by Isidora Morganach, represents hyper-compassionate control, characterized by intolerance of suffering, experiential avoidance, elimination of negative affect, moral certainty, and paternalistic intervention. Between these poles, the model includes an intermediate zone of proportionate psychiatric care, informed by emotional integration, psychological flexibility, shared decision-making, clinical humility, contextual judgment, and ethical proportionality. This middle space represents proportionate, reflective, collaborative, context-sensitive, and ethically bounded intervention. Both extreme pathways converge on the broader concept of power exercised without emotional integration. In contemporary psychiatric practice, this may correspond to risks such as psychiatric overreach, overmedicalization, erosion of therapeutic boundaries, and loss of clinical humility.

Taken together, these two narrative pathways provide the basis for the conceptual model proposed in this review. The model does not imply diagnostic equivalence between fictional characters and clinical populations; rather, it organizes the narrative-clinical parallels identified above into two extreme forms of psychiatric overreach: destructive domination and hyper-compassionate control. The model also includes an intermediate zone, representing proportionate psychiatric care. This middle position is not an absence of intervention, but an ethically disciplined form of intervention guided by emotional integration, autonomy, clinical humility, contextual judgment, and proportionality. Accordingly, the model should be read as a heuristic continuum rather than as a categorical classification: sources, clinicians, patients, or interventions are not assigned to one character or the other, but are interpreted according to the conceptual risks and safeguards they illuminate. **Figure 1** summarizes this framework, showing how both pathways emerge from power exercised without sufficient emotional integration and may converge on boundary erosion, overmedicalization, and loss of clinical humility, while the intermediate zone represents the reflective space in which care can remain therapeutic without becoming controlling.

## 4 Discussion

The Results section organizes the proposed model around three elements. First, the destructive domination pathway shows how grandiosity, emotional suppression, moral disengagement, and coercive authority may converge when power is detached from vulnerability and empathy. Second, the hyper-compassionate control pathway shows how distress intolerance, experiential avoidance, paternalism, medicalization, and excessive intervention may allow benevolent motives to exceed ethical boundaries. Third, the intermediate zone of proportionate psychiatric care highlights the importance of emotional integration, autonomy, shared decision-making, contextual judgment, ethical proportionality, and clinical humility. These elements provide the basis for the clinical, ethical, and educational implications discussed below.

### 4.1 What fictional extremes reveal about psychiatric practice

This narrative review used the *Wizarding World* to examine how power may exceed its therapeutic mandate when insufficiently constrained by emotional integration, ethical judgment, and clinical humility. This approach is consistent with a broader movement in contemporary psychiatry and medical humanities that recognizes cultural narratives as useful tools for examining clinical authority, professional identity, and the meanings attached to suffering. The comparison between Lord Voldemort and Isidora Morganach does not seek to diagnose fictional characters. Rather, it uses two narrative extremes to clarify a real problem in contemporary psychiatry: the difficulty of distinguishing care from control when the capacity to intervene continues to expand. The *Wizarding World* was therefore not treated as an object of literary analysis, but as a structured narrative space through which contemporary psychiatric tensions could be explored.

The two figures illuminate opposite, but clinically relevant, distortions of authority. Voldemort represents power organized around domination, grandiosity, and suppression of vulnerability. His narrative arc illustrates the destructive consequences of refusing dependency, fear, and human limitation. Isidora, by contrast, represents power organized around relief, compassion, and the desire to eliminate suffering. Her arc is more unsettling from a clinical perspective because it begins with benevolent intent. She does not aim to terrorize; she aims to heal. Yet the result is still a form of overreach, because suffering is treated not as an experience to be understood or integrated, but as a defect to be removed.

This distinction is central to the argument of the present review. Psychiatric overreach should not be imagined only as coercion, institutional power, or authoritarian control. These forms remain important, and the history of psychiatry provides many reasons to remain vigilant about them. However, overreach may also arise through softer mechanisms: excessive risk aversion, diagnostic expansion, premature intervention, moral certainty, and the conversion of painful but meaningful experiences into targets for technical correction (13, 15, 16, 42, 52). In this sense, the *Wizarding World* does not merely dramatize the misuse of power; it also dramatizes the more ambiguous possibility that care itself may become excessive when it is detached from restraint.

The figure of Isidora is therefore crucial. Without her, the manuscript would risk making a familiar point about grandiosity, domination, and narcissistic power. Her storyline allows the analysis to move into more difficult ethical terrain. It asks whether the desire to relieve suffering can itself become harmful when it assumes that all distress is pathological, unnecessary, or intolerable. This is a particularly relevant question for psychiatry, a discipline whose legitimacy rests on its ability to reduce suffering, but whose boundaries depend on not confusing suffering with disorder in every instance.

### 4.2 Emotional regulation as an ethical boundary

One of the central implications of this review is that emotional regulation is not only a psychological construct, but also an ethical boundary. Contemporary models define emotion regulation as the modulation of emotional experience, expression, and physiological response in ways that may support adaptive functioning (24–26). This is different from emotional elimination. The clinical goal is rarely, if ever, the abolition of sadness, grief, fear, or anger. Rather, psychiatry and psychotherapy aim to reduce suffering when it becomes disabling, disproportionate, persistent, or destructive, while preserving the person’s capacity to experience, interpret, and integrate emotional life.

The literature on emotion regulation supports this distinction. Rigid suppression has been associated with poorer mental health outcomes across diagnostic groups, while flexible regulation and psychological flexibility are associated with better adaptation (27–29). More recent work continues to conceptualize emotional dysregulation as a transdiagnostic process across psychiatric conditions (53). In addition, emerging evidence links difficulties in emotion regulation with traits relevant to psychopathy and antagonistic personality functioning, reinforcing the importance of affective regulation for interpersonal and moral behavior (54). In this context, Voldemort and Isidora can be understood as symbolic extremes of dysregulated power: one suppresses vulnerability and externalizes fear through domination; the other cannot tolerate suffering and attempts to remove it altogether.

The ethical significance of this distinction becomes especially clear in clinical practice. A clinician who cannot tolerate a patient’s distress may feel compelled to act too quickly, diagnose too readily, medicate too reflexively, hospitalize too defensively, or reassure too prematurely. These actions may sometimes be necessary and beneficial. However, when driven primarily by the clinician’s inability to remain with uncertainty or suffering, they may become forms of emotional overcontrol. In that sense, Isidora’s error is not simply magical or fictional; it is an exaggerated version of a familiar clinical temptation.

Psychiatric care requires the capacity to bear witness to distress without immediately converting it into an object of control. This does not mean romanticizing suffering or minimizing severe mental illness. On the contrary, it means taking suffering seriously enough to ask what kind of response it requires. Some distress requires urgent intervention. Some requires treatment over time. Some requires social support, meaning-making, mourning, protection, or practical change. Some must be accompanied before it can be transformed. Emotional regulation, then, is not merely a patient skill; it is also a professional discipline.

### 4.3 Moral authority, medicalization, and the risk of overreach

Building on the emotional boundary described above, this section shifts from the regulation of distress to the ethical use of clinical authority. The question is no longer only how clinicians understand suffering, but how psychiatric authority translates that understanding into diagnosis, intervention, and decisions about care. The concept of moral authority is unavoidable in psychiatry. Unlike many areas of medicine, psychiatry often deals directly with judgment, agency, risk, autonomy, identity, and social functioning. Psychiatric decisions may affect not only symptoms, but also self-understanding, relationships, education, work, legal status, and freedom. This makes psychiatric authority both necessary and dangerous. It is necessary because severe mental disorders can profoundly impair judgment, safety, and functioning. It is dangerous because psychiatric categories and interventions carry social meaning beyond the clinic.

Debates about medicalization and overdiagnosis remain highly relevant here. Wakefield’s harmful dysfunction model attempted to distinguish disorder from socially disvalued but non-disordered forms of suffering (16). Horwitz and Wakefield later argued that psychiatry risks transforming normal sorrow into depressive disorder when contextual and proportional features of sadness are insufficiently considered (13). More recent discussions have extended these concerns to overdiagnosis in psychiatry broadly, including the difficulty of separating clinical disorder from variation in thought, emotion, attention, and behavior (15, 52). In child and adolescent psychiatry, debates about ADHD diagnosis show how the boundaries between impairment, variation, service access, and overdiagnosis can become clinically and politically complex (17).

The present review does not argue against diagnosis or intervention. Such a position would be clinically irresponsible and inconsistent with the evidence supporting psychiatric care. Rather, it argues that psychiatric practice requires proportionality. The risk lies not in intervention itself, but in intervention that loses sight of context, autonomy, uncertainty, and the adaptive functions of emotional experience. Isidora’s story offers a useful metaphor for this risk: once suffering is defined as something that should not exist, the person who claims the power to remove it may also claim authority over the inner life of another.

This is where moral authority may become overreach. In a paternalistic model, the clinician determines what is best for the patient and acts accordingly, sometimes with limited attention to the patient’s own values or interpretation of their experience. Emanuel and Emanuel’s classic typology of physician-patient relationships distinguished paternalistic, informative, interpretive, and deliberative models, emphasizing that clinical authority can be organized in different ways (38). Psychiatry, perhaps more than most specialties, must continually negotiate among these models. Acute risk, psychosis, severe depression, mania, and impaired decision-making may require directive care. But outside such circumstances, moral certainty can become clinically hazardous.

The challenge is therefore not to abandon authority, but to discipline it. Psychiatric authority should be guided by evidence, proportionality, respect for autonomy, and awareness of uncertainty. It should also be reflexive: the clinician must ask not only “Can I reduce this suffering?” but also “What kind of suffering is this?”, “What does it mean to this person?”, “What would be lost if it were removed too quickly?”, and “Whose discomfort is driving the intervention?”. These questions are not decorative. They are safeguards against turning care into control.

### 4.4 Clinical humility and the limits of technical power

The concept of humility is often invoked in medicine, but it is particularly important in psychiatry because the field operates at the intersection of biology, subjectivity, culture, and social norms. Epistemic humility refers to awareness of the limits of one’s knowledge, including the possibility that diagnostic categories, clinical formulations, and risk assessments may be incomplete or mistaken. Recent work on relational epistemic humility emphasizes that humility is not only an internal attitude, but also something enacted within the clinical encounter through listening, openness, and responsiveness to the patient’s perspective (51).

This is directly relevant to the present framework. Voldemort lacks humility because he cannot acknowledge limitation. Isidora lacks humility because she cannot acknowledge the limits of her own benevolence. Both forms matter clinically. A clinician may overreach through certainty of power, but also through certainty of goodness. The second form is harder to detect because it may appear compassionate, progressive, or therapeutic. Yet benevolent certainty can still override patient agency.

Diagnostic error literature also supports the need for humility. Mental health diagnosis is complex, and errors may arise from overlapping symptoms, comorbidity, contextual ambiguity, stigma, time pressure, and system-level constraints (55). These challenges do not invalidate diagnosis; they make careful diagnosis more important. A humble psychiatry is not a weak psychiatry. It is a psychiatry that recognizes the strength of disciplined uncertainty.

Technical power must therefore be accompanied by interpretive restraint. Psychopharmacology, neuromodulation, digital phenotyping, predictive analytics, and early intervention strategies will likely continue to expand psychiatric capacity. These tools can improve care, but they may also intensify the temptation to intervene earlier, broader, and more aggressively. The ethical question is not whether psychiatry should use powerful tools. It should. The question is whether the field can preserve humility while using them.

In this regard, the *Wizarding World* offers a memorable warning: power becomes dangerous not only when it is cruel, but also when it becomes too certain of its own necessity. A mature psychiatry must therefore resist both fantasies: the fantasy of total control and the fantasy of total relief.

### 4.5 Implications for psychiatric education and training

The framework proposed in this review has practical implications for psychiatric education. Cultural narratives can create psychologically safe spaces in which trainees examine difficult ethical questions without immediately becoming defensive. The use of narrative in medicine has been associated with reflection, empathy, professional identity formation, and attention to patient experience (10, 11, 56). At the same time, narrative medicine has limitations and should not be treated as automatically patient-centered or ethically sufficient (57). Its value depends on how carefully it is used.

The *Wizarding World* may be particularly useful because its moral universe is familiar to many learners, but complex enough to support serious discussion. Voldemort allows discussion of domination, dehumanization, grandiosity, and fear of vulnerability. Isidora allows discussion of compassion, paternalism, emotional overcontrol, and the ethics of relieving suffering. Together, they help trainees see that the problem of power in psychiatry is not one-dimensional.

Several educational applications are possible. First, the model could be used in seminars on psychiatric ethics to explore proportionality, autonomy, paternalism, and coercion. Second, it could be used in psychotherapy training to distinguish emotional regulation from avoidance or suppression. Third, it could support reflective exercises on clinicians’ emotional responses to patient distress. Fourth, it could be integrated into discussions about overdiagnosis and medicalization, particularly in areas where diagnostic thresholds are contested or where access to support depends heavily on receiving a label.

Such teaching should be explicit that fictional characters are not clinical cases. The goal is not to ask whether Voldemort has narcissistic personality disorder or whether Isidora has a psychiatric diagnosis. That would flatten the analysis and risk trivialization. The more valuable question is what these characters reveal about the temptations of power: the temptation to dominate what one fears, and the temptation to eliminate what one cannot bear.

### 4.6 Implications for contemporary psychiatric practice

For contemporary psychiatric practice, the main implication is that intervention should be guided by both clinical evidence and ethical proportionality. The ability to reduce distress is one of psychiatry’s greatest strengths. But distress is not a single phenomenon. It may be symptomatic, traumatic, relational, developmental, existential, social, or moral. Treating all distress as equivalent risks narrowing the clinical imagination and may obscure the distinction between disorder, understandable suffering, and contextual adversity (13, 16, 37). This is the clinical meaning of the intermediate zone proposed in the model: psychiatric care should neither avoid intervention when suffering is severe nor convert all distress into an object of control. Instead, it should remain proportionate, collaborative, context-sensitive, and attentive to the patient’s values and agency.

A proportionate psychiatric response should therefore ask several questions. Is the distress part of a diagnosable disorder? Is it impairing function? Is it persistent, escalating, or dangerous? Is it understandable within context? What does the patient want? What are the risks of action and inaction? What alternatives exist beyond immediate medical intervention? These questions do not slow care unnecessarily; they refine it. They are also consistent with shared decision-making approaches, which emphasize collaboration between clinicians and patients, integration of best available evidence, and attention to the patient’s preferences and values (49, 50).

The model also reminds clinicians to monitor their own emotional responses. The wish to rescue can be powerful, especially in the face of severe suffering, and psychotherapy literature has described rescue fantasies as meaningful features of therapists’ professional relational narratives (47**).** Yet rescue fantasies may lead to excessive intervention when not balanced by patient agency and clinical humility. Conversely, fear-driven responses may lead to coercion, defensive practice, and overmanagement of risk. Coercive practices in psychiatry raise persistent ethical concerns because they restrict autonomy and may produce harm even when intended to prevent danger (18, 19, 58). Both patterns (the impulse to rescue and the impulse to control) can be understood as forms of power without emotional integration.

This argument is especially relevant in an era of expanding psychiatric technologies. As tools become more powerful, the ethical burden increases. Early detection, digital monitoring, neuromodulation, and pharmacological innovation may offer major benefits, but they also require careful attention to consent, proportionality, uncertainty, privacy, bias, and meaning (20–23). The future of psychiatry should not be a choice between under-treatment and overreach. It should be a disciplined practice of knowing when to act, how much to act, and when to accompany rather than control.

### 4.7 Limitations

This review has several limitations. First, it is a narrative and interpretative synthesis rather than a systematic review or meta-analysis. Although structured search and evidence summarization procedures were used, selection bias cannot be excluded. No formal risk-of-bias assessment, evidence grading, or pooled effect estimation was performed. Second, the analysis relies on fictional material, and fictional characters should not be treated as clinical subjects. The framework is therefore conceptual rather than diagnostic.

A related methodological limitation concerns the status of narrative itself. Narrative medicine and the medical humanities can help clinicians examine meaning, identity, suffering, and ethical tension, but narrative should not be treated as a universal or inherently superior form of understanding. Critiques of narrativity have cautioned that not all persons experience themselves primarily through coherent life stories, and that excessive emphasis on narrative coherence may obscure fragmentation, silence, contradiction, or forms of suffering that resist narrative organization (44, 45). Accordingly, this review does not assume that fictional narrative offers direct access to clinical truth. Rather, narrative is used as a reflective and pedagogical framework that can illuminate selected ethical tensions while remaining limited, partial, and dependent on interpretation.

Third, the interpretation of the *Wizarding World* is culturally situated. Readers from different backgrounds may interpret the same characters and narrative events differently. Fourth, the inclusion of Isidora Morganach from *Hogwarts Legacy* introduces material from the expanded transmedia universe rather than the original novels. Although this was explicitly acknowledged, some readers may view the primary literary canon and later licensed works as analytically distinct. Finally, the concept of psychiatric overreach is itself contested. Concerns about overdiagnosis and medicalization must be balanced against the ongoing reality of underdiagnosis, undertreatment, stigma, and lack of access to care in many settings.

These limitations do not undermine the conceptual value of the framework, but they define its scope. The model should be understood as a tool for reflection, teaching, and ethical analysis, not as an empirical taxonomy.

## 5 Conclusion

The *Wizarding World* offers more than a familiar cultural setting. It provides a narrative structure through which psychiatry can examine its own relationship to power. Voldemort and Isidora Morganach represent two contrasting forms of power without emotional integration. One seeks domination by rejecting vulnerability; the other seeks relief by eliminating suffering. One is cruel; the other is compassionate. Yet both show how power may become dangerous when it is not limited by emotional integration, clinical humility, and respect for the autonomy of others.

The proposed model suggests that the ethical task of psychiatry lies in the intermediate zone between these extremes. This zone is not defined by inaction, but by proportionate, reflective, collaborative, and context-sensitive care. For psychiatry, the lesson is therefore not that intervention is dangerous in itself. On the contrary, psychiatric intervention can be lifesaving, liberating, and profoundly humane. The lesson is that intervention must remain clinically justified, ethically bounded, and attentive to the meanings that distress holds for each person.

The task of psychiatry is not to abolish all distress, nor to exercise authority without uncertainty. Its task is to relieve suffering while preserving the human meanings through which suffering is understood, endured, and transformed.
