# Supplementary material for: Power without limits: exploring emotional regulation, moral authority, and psychiatric overreach through the Wizarding World of Harry Potter
Source: Front Psychiatry. 2026 Jul 8;17:1889488. doi: 10.3389/fpsyt.2026.1889488 (PMC13388311; doi:10.3389/fpsyt.2026.1889488)
Supplement: Supplementary file 1 [file Table1.docx]

**Supplementary Table 1**

*Search strings used in the narrative review*

| **Conceptual domain** | **Search string** |
| --- | --- |
| Wizarding World and mental health | (“Harry Potter” OR “Wizarding World” OR “Hogwarts Legacy”) AND (psychiatry OR psychology OR psychotherapy OR “mental health” OR recovery OR “medical humanities” OR “narrative medicine”) |
| Fiction, narrative, and psychiatry | (fiction OR literature OR narrative OR “fictional character*” OR “cultural narrative*”) AND (psychiatry OR psychology OR psychotherapy OR “mental health” OR ethics OR “medical humanities” OR “narrative medicine”) |
| Emotional regulation and psychological flexibility | (“emotion regulation” OR “emotional regulation” OR “emotion suppression” OR “emotional suppression” OR “psychological flexibility” OR “experiential avoidance” OR “distress tolerance”) AND (psychiatry OR psychopathology OR “mental disorder*” OR psychotherapy OR “mental health”) |
| Narcissism, domination, and Dark Triad traits | (narcissism OR “pathological narcissism” OR grandiosity OR dominance OR “Dark Triad” OR psychopathy OR Machiavellianism OR antagonism) AND (emotion OR “emotion regulation” OR empathy OR coercion OR violence OR “interpersonal functioning”) |
| Medicalization, overdiagnosis, and psychiatric overreach | (medicalization OR overmedicalization OR overdiagnosis OR “diagnostic expansion” OR “psychiatric overreach” OR “excessive intervention”) AND (psychiatry OR “mental disorder*” OR diagnosis OR treatment OR ethics) |
| Ethics, paternalism, and clinical authority | (paternalism OR autonomy OR beneficence OR coercion OR “clinical authority” OR “medical authority” OR “shared decision-making” OR “informed consent”) AND (psychiatry OR “mental health care” OR psychotherapy OR ethics) |
| Digital monitoring and early intervention | (“digital phenotyping” OR “digital monitoring” OR “early intervention” OR “clinical high risk” OR “risk prediction”) AND (psychiatry OR “mental health” OR ethics OR consent OR privacy OR stigma OR proportionality) |
| Narrative medicine and medical humanities | (“narrative medicine” OR “medical humanities” OR “illness narrative*” OR “reflective writing” OR “narrative psychiatry”) AND (psychiatry OR psychotherapy OR ethics OR empathy OR “clinical education”) |
